# Supplementary material for: An empirical energy landscape reveals mechanism of proteasome in polypeptide translocation
Source: eLife. 2022 Jan 20;11:e71911. doi: 10.7554/eLife.71911 (PMC8853663; doi:10.7554/eLife.71911)
Supplement: Figure 4—source data 3. — Related to Figure 4C and D. [file elife-71911-fig4-data3.pdf]

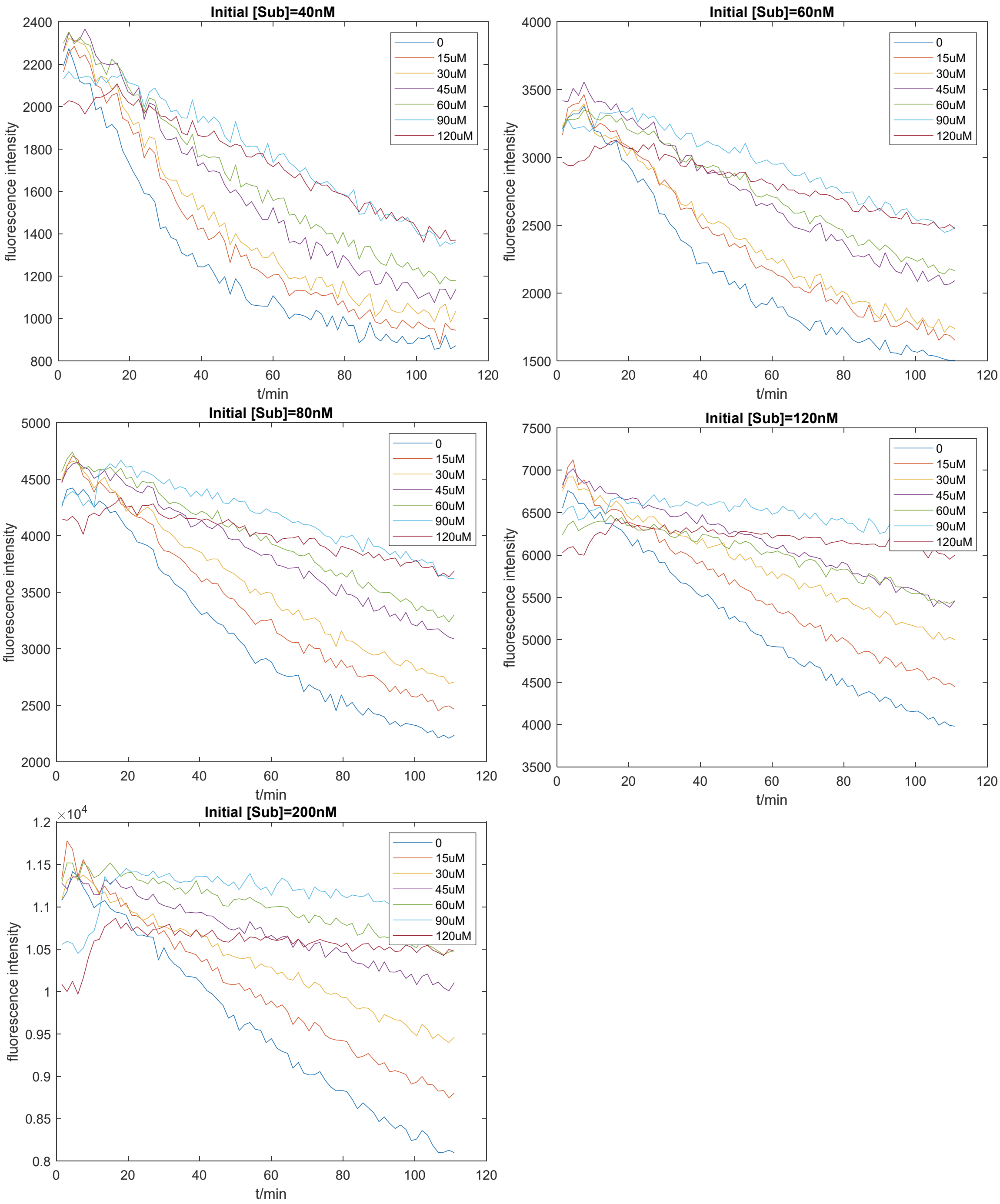

Source data related to figure 4C,D. Ubiquitylated cycB-iRFP (sub) at indicated concentrations was incubated with purified 26S proteasome in the presence of 500uM ATP plus various concentrations of ATP-γS. The fluorescence intensity from iRFP was monitored using a plate reader. Each trace is an average of three replica.
