# Supplementary material for: An empirical energy landscape reveals mechanism of proteasome in polypeptide translocation
Source: eLife. 2022 Jan 20;11:e71911. doi: 10.7554/eLife.71911 (PMC8853663; doi:10.7554/eLife.71911)
Supplement: Figure 5—source data 1. — Related to Figure 5A and B. [file elife-71911-fig5-data1.pdf]

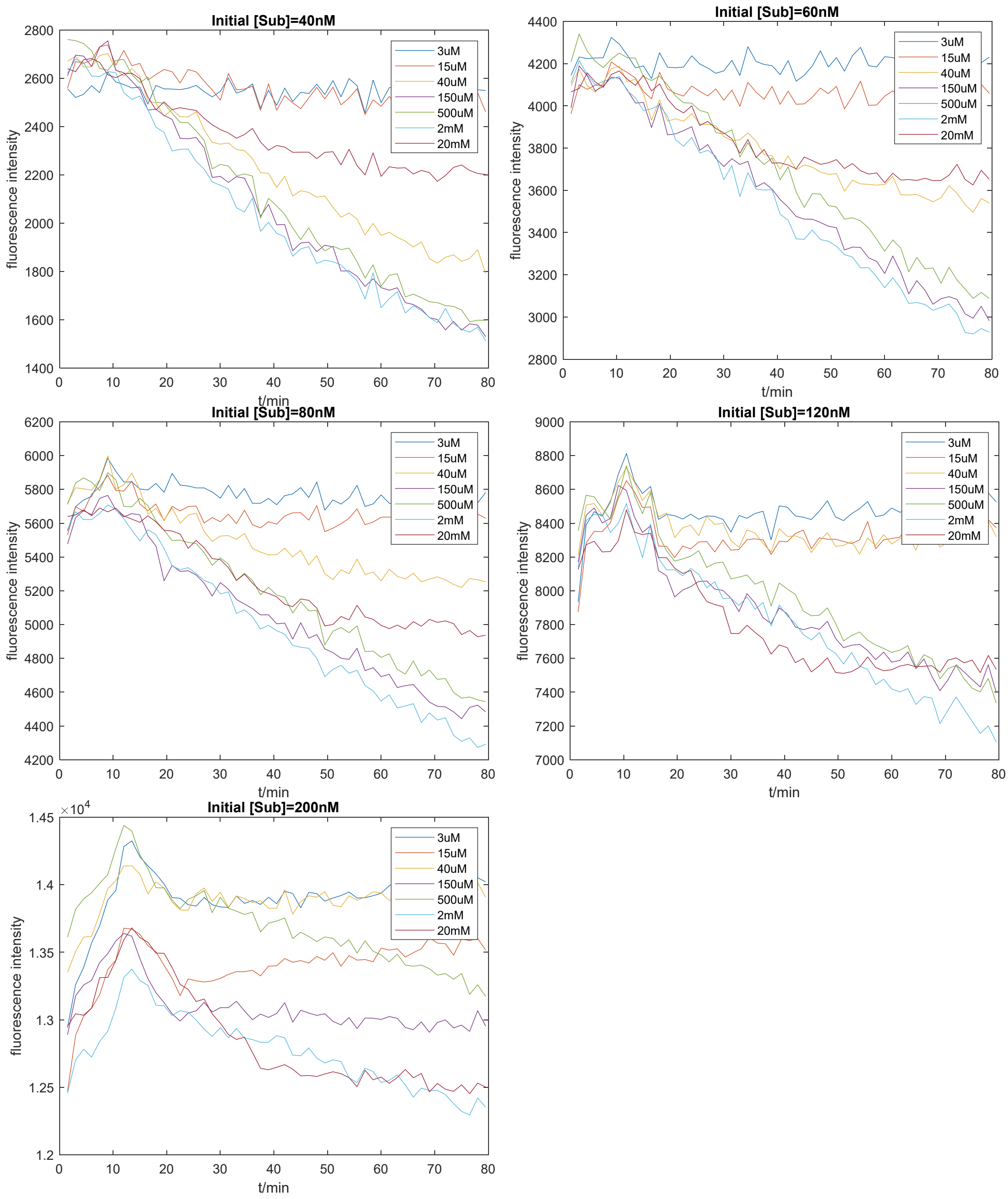

Source data related to figure 5A,B. Ubiquitylated cycB-DHFR-iRFP (sub) at indicated concentrations was incubated with purified 26S proteasome in the presence of various concentrations of ATP without folic acid. The fluorescence intensity from iRFP was monitored using a plate reader. Each trace is an average of three replica.
