# Supplementary material for: An empirical energy landscape reveals mechanism of proteasome in polypeptide translocation
Source: eLife. 2022 Jan 20;11:e71911. doi: 10.7554/eLife.71911 (PMC8853663; doi:10.7554/eLife.71911)
Supplement: Figure 5—source data 2. — Related to Figure 5A and B. [file elife-71911-fig5-data2.pdf]

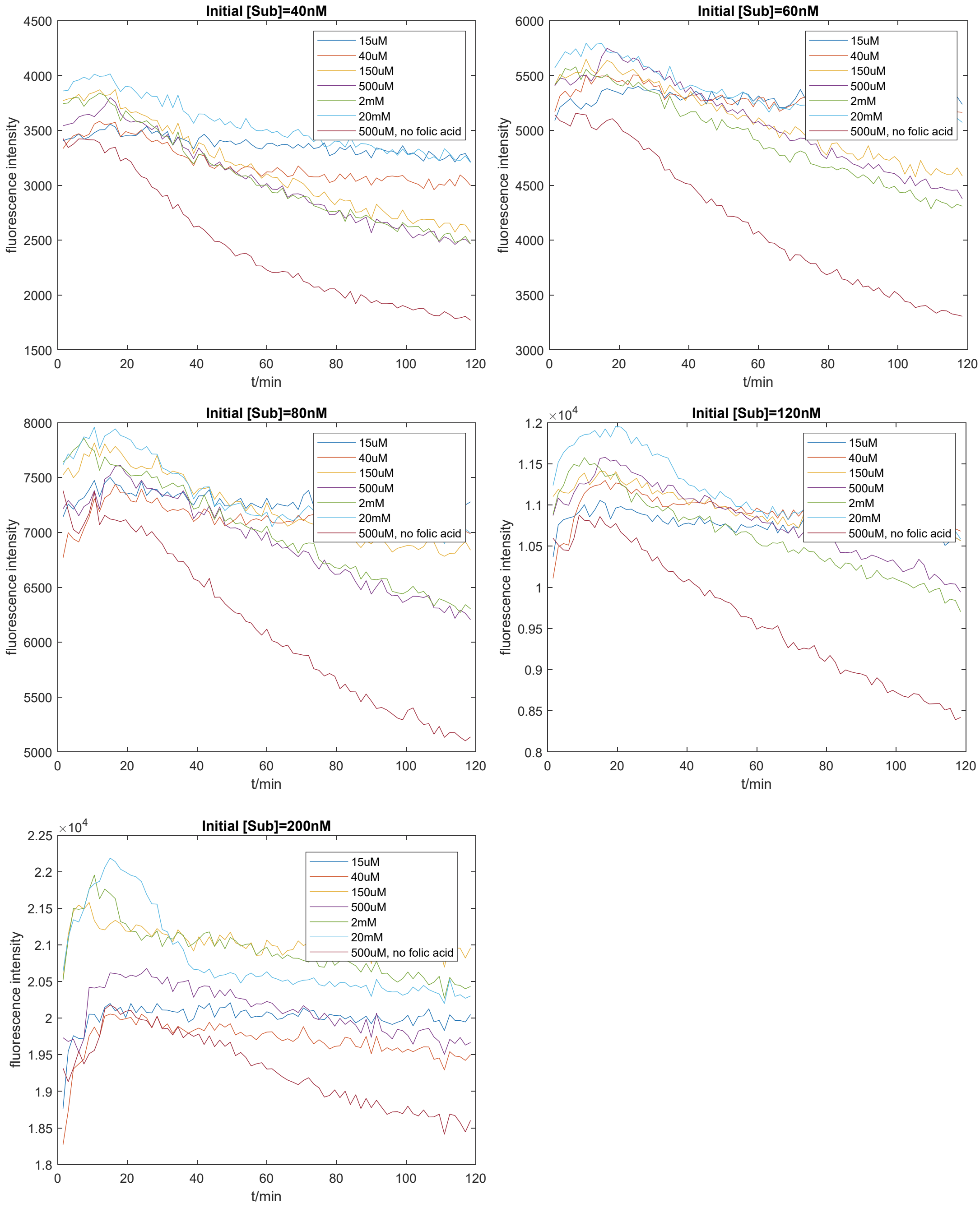

Source data related to figure 5A,B. Ubiquitylated cycB-DHFR-iRFP (sub) at indicated concentrations was incubated with purified 26S proteasome in the presence of various concentrations of ATP and 0.8mM folic acid. The fluorescence intensity from iRFP was monitored using a plate reader. Each trace is an average of three replica.
