# Supplementary material for: An empirical energy landscape reveals mechanism of proteasome in polypeptide translocation
Source: eLife. 2022 Jan 20;11:e71911. doi: 10.7554/eLife.71911 (PMC8853663; doi:10.7554/eLife.71911)
Supplement: Figure 5—source data 3. — Related to Figure 5F. [file elife-71911-fig5-data3.pdf]

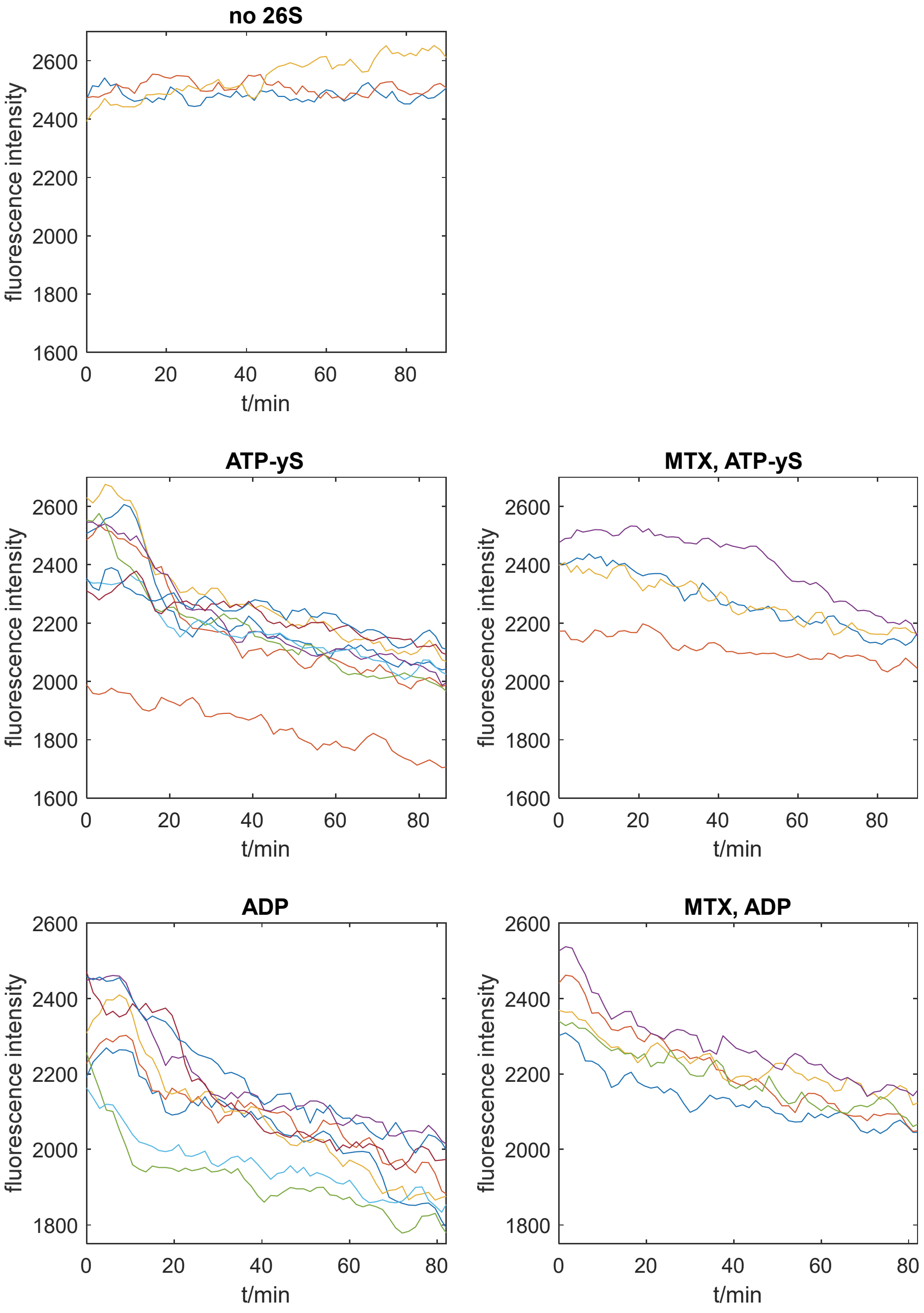

Source data related to figure 5F. Ubiquitylated cycB-iRFP was incubated with purified 26S proteasome in the presence of ubiquitylated cycB-DHFR-iRFP$^{(\text{Dark})}$ as a competitor. The buffer contains 500µM ATP mixed with either ADP or ATP-γS. Methotrexate (MTX) was added as indicated. Each trace is an independent replicate.
